# Immunostimulatory Properties of Chemotherapy in Breast Cancer: From Immunogenic Modulation Mechanisms to Clinical Practice

**DOI:** 10.3389/fimmu.2021.819405

**Published:** 2022-01-05

**Authors:** Jinguo Zhang, Shuaikang Pan, Chen Jian, Li Hao, Jie Dong, Qingqing Sun, Hongwei Jin, Xinghua Han

**Affiliations:** Department of Medical Oncology, The First Affiliated Hospital of USTC, Division of Life Science and Medicine, University of Science and Technology of China, Hefei, China

**Keywords:** breast cancer, chemotherapy, immunotherapy, immunogenic modulation, clinic trial

## Abstract

Breast cancer (BC) is the most common malignancy among females. Chemotherapy drugs remain the cornerstone of treatment of BC and undergo significant shifts over the past 100 years. The advent of immunotherapy presents promising opportunities and constitutes a significant complementary to existing therapeutic strategies for BC. Chemotherapy as a cytotoxic treatment that targets proliferation malignant cells has recently been shown as an effective immune-stimulus in multiple ways. Chemotherapeutic drugs can cause the release of damage-associated molecular patterns (DAMPs) from dying tumor cells, which result in long-lasting antitumor immunity by the key process of immunogenic cell death (ICD). Furthermore, Off-target effects of chemotherapy on immune cell subsets mainly involve activation of immune effector cells including natural killer (NK) cells, dendritic cells (DCs), and cytotoxic T cells, and depletion of immunosuppressive cells including Treg cells, M2 macrophages and myeloid-derived suppressor cells (MDSCs). Current mini-review summarized recent large clinical trials regarding the combination of chemotherapy and immunotherapy in BC and addressed the molecular mechanisms of immunostimulatory properties of chemotherapy in BC. The purpose of our work was to explore the immune-stimulating effects of chemotherapy at the molecular level based on the evidence from clinical trials, which might be a rationale for combinations of chemotherapy and immunotherapy in BC.

## Introduction

Breast cancer (BC), a highly heterogeneous disease, is the most common cancer among women (1). The 2021 global cancer statistics showed about 2.3 million newly diagnosed BC and approximately 0.69 million BC deaths, with a higher incidence than lung cancer (2, 3). The survival rates of BC vary widely worldwide, with an estimated five-year survival rate of 80% in developed countries while less than 40% in developing countries (1, 4). BC is generally comprised of luminal A, luminal B, HER2 overexpression, basal-like triple negative breast cancer (TNBC), and other special subtypes proposed by St. Gallen International Breast Cancer Conference in 2013 (5). Subtype identification provides a fundamental basis for decision making in the therapeutic management of BC (6). Thus, to select the most appropriate systemic therapy for BC, subtype classification is quite necessary (7). Modern therapy of BC involves a combination of surgery of operable tumors, chemotherapy (neoadjuvant/adjuvant), endocrine therapy, targeted therapy, radiotherapy and immunotherapy (8). The initial approach for BC was aggressive surgery in the early 20th century (6). And the types of chemotherapy and their indications have experienced rapid growth since radical mastectomy evolved from more aggressive to less aggressive (9). In 2001, a National Institute of Health consensus panel concluded that owing to a clear survival benefit by adjuvant polychemotherapy, it should be recommended to the majority of women with localized BC regardless of lymph node, menopausal, or hormone receptor status (10). Since then, the status of chemotherapy in the treatment of BC has been established.

It is traditionally recognized that BC is characterized by low tumor mutation burden (TMB) and poorly immunogenic. However, recent evidence revealed that infiltrating lymphocytes (TILs) and programmed cell death-ligand 1 (PD-L1) were expressed in a considerable proportion of HER2+ BC and TNBC patients (11). Cancer immunotherapy aims to provoke an immune response by either enhancing the cytotoxic potential of immune cells or blocking the immunosuppressive tumor microenvironment (12). Immunotherapy has a rich content including immune checkpoint blockade, adoptive cell therapies, adoptive cell therapies vaccines and oncolytic viruses (13). Among these therapy strategies, the United States Food and Drug Administration (FDA) has approved immune checkpoint inhibitors (ICIs) targeting PD-1 (programmed cell death receptor 1), PD-L1 (programmed cell death 1 ligand 1), and CTLA-4 (cytotoxic T-lymphocyte-associated antigen-4) for treatment of solid tumors such as BC (14, 15). Among all subtype of BC, TNBC, the most invasive BC, was regarded as the most immunogenic type due to the presence of tumor neoantigens, and high levels of lymphocytic infiltration, mutation (16). The results of the IMpassion130 trial demonstrated a substantial overall survival (OS) benefit and brought BC into immunotherapy era (17). Thus, considerable effort has been dedicated to combination of standard-of-care chemotherapies with immunotherapy in BC.

Chemotherapy was previously thought to be solely immunosuppressive, but recent data showed that it might also possess immunostimulatory properties. In this mini review, we summarized the updated clinical trials on immunotherapy and chemotherapy combinations in BC. More importantly, we discussed recent literature on the immunomodulatory effects of chemotherapy with a focus on immunostimulatory function.

## Immune Checkpoint Inhibitors Combined With Chemotherapy in BC

First, the IMpassion130 (NCT02425891) trial funded by F. Hoffmann–La Roche/Genentech comparing chemotherapy plus placebo versus chemotherapy plus atezolizumab brought BC into the immunotherapy era. In this phase 3 trial, 902 patients with untreated metastatic TNBC were randomly assigned (in a 1:1 ratio) to receive atezolizumab plus nab-paclitaxel or placebo plus nab-paclitaxel. Patients received atezolizumab 840mg or placebo intravenously on days 1 and 15 and received nab-paclitaxel at a dose of 100 mg/m^2^ that administered intravenously on days 1, 8, and 15 of every 28-day cycle. This trial displayed a substantial progression-free survival (PFS) benefit in patients with metastatic TNBC either the intention-to-treat population or the PD-L1–positive subgroup. With a median follow-up of 12.9 months, among the ITT population, the median PFS was significantly prolonged after the addition of atezolizumab as compared to chemotherapy alone (7.2 vs 5.5 months); further, in the PD-L1 positive population, the respective PFS benefit was more improved (7.5 vs 5.0 months). Regarding the intention-to-treat analysis, the median OS was 21.3 months (atezolizumab plus nab-paclitaxel) and 17.6 months (placebo plus nab-paclitaxel), while in the PD-L1 positive population, the OS was increased 9.5 months with the addition of atezolizumab (25.0 vs. 15.5 months) (18). The above data has attracted significant interest in clinical scientist, and then a series of ongoing trials that were design for chemotherapy combined with immunotherapy begun to emerge. Subsequent randomized Phase III trial IMpassion131 (NCT03125902) evaluated first-line paclitaxel with or without atezolizumab for unresectable locally advanced/metastatic TNBC. 651 eligible patients were randomized 2:1 to atezolizumab plus paclitaxel or placebo plus paclitaxel. At the primary analysis, no significant improvement of PFS or OS was observed while adding atezolizumab to paclitaxel and the reasons for this remain unclear. At a median follow-up of 9.0 months (atezolizumab-paclitaxel arm) and 8.6 months (placebo-paclitaxel arm), in the PD-L1-positive population, median PFS was 6.0 months and 5.7 months, respectively. Final OS results also showed no difference between arms (atezolizumab-paclitaxel arm 22.1 months versus placebo-paclitaxel arm 28.3 months). Results in the ITT population were in accord with the PD-L1-positive population. Conclusions from IMpassion131 also contrasted with results from the KEYNOTE-355 trial (we will further elaborate below) that evaluated a more extensively chemotherapy backbones (including both paclitaxel and nab-paclitaxel, as well as gemcitabine/carboplatin) with a different immunotherapy agent, pembrolizumab (15). Both IMpassion130 and IMpassion131 excluded patients with early relapse (disease progression within 12 months of chemotherapy for early breast cancer), however IMpassion132 (NCT03371017) is one of the first trials prospectively focusing on the early relapsing TNBC population. The IMpassion132 trial combined atezolizumab with two commonly used non-taxane chemotherapy regimens (gemcitabine plus carboplatin, or single-agent capecitabine), which aimed to determine whether similar improvement observed in the IMpassion130 could be achieved with an alternative chemotherapy backbone in the case of early relapse. This phase III trial is ongoing and the primary end point is OS in the ITT population (19).

KEYNOTE-355 (NCT02819518), compared pembrolizumab plus chemotherapy (nab-paclitaxel; paclitaxel; or gemcitabine plus carboplatin) with placebo plus chemotherapy, showed a significant and clinically meaningful improvement in PFS among patients with locally recurrent inoperable or metastatic TNBC with combined positive score(CPS)of 10 or more. Pembrolizumab combined chemotherapy showed a positive result both in patients CPS≥10 and CPS≥1. Median PFS was 9·7 months and 5·6 months (pembrolizumab–chemotherapy and placebo–chemotherapy, respective) among patients with CPS≥10. Among patients with CPS≥1, median PFS was 7·6 and 5·6 months. Results in the ITT population were 7·5 and 5·6 months. These findings suggested a role for the combination of pembrolizumab and chemotherapy for the first-line treatment of metastatic TNBC (20). Compared to KEYNOTE-355, another ongoing phase III clinical trial KEYNOTE-522 (NCT03036488) mainly focused on patients with early TNBC. A pathological complete response (pCR) at the time of definitive surgery and event-free survival (EFS) in the ITT population were the two primary end points. A total of 1174 patients with previously untreated stage II or stage III TNBC were randomly assigned (in a 2:1 ratio) to the pembrolizumab–chemotherapy group (784 patients) or the placebo–chemotherapy group (390 patients). Patients in pembrolizumab–chemotherapy group received therapy with pembrolizumab plus paclitaxel and carboplatin. Placebo–chemotherapy group received placebo plus paclitaxel and carboplatin, and both groups received doxorubicin–cyclophosphamide or epirubicin–cyclophosphamide. At the first interim analysis of 602 patients, the percentage of patients with a pCR was 64.8% (pembrolizumab–chemotherapy group) and 51.2% (placebo–chemotherapy group). In the PD-L1–positive population, the percentage of patients with a pCR was 68.9% versus 54.9% (pembrolizumab–chemotherapy group versus placebo–chemotherapy group), while the percentage of patients with a pCR was 45.3% versus 30.3% (pembrolizumab–chemotherapy group versus placebo–chemotherapy group) in the PD-L1–negative population. The patients who received pembrolizumab showed a significantly higher pathological complete response percentage than those who received placebo. Across all treatment phases, the incidence of treatment-related adverse events of grade 3 or higher was 78.0% and 73.0%, including death in 0.4% (3 patients) and 0.3% (1 patient), in the pembrolizumab–chemotherapy group and placebo–chemotherapy group, respectively (21).

The above clinical trials including chemotherapy plus atezolizumab or pembrolizumab not only provide powerful evidence for the benefits of chemotherapy combined with immunotherapy, but also provide us new treatment alternatives, which enable more BC patients to benefit from immunotherapy. Several clinical trials have been designed to explore the potentiality of chemotherapy combined with immunotherapy with a variety of patterns. I-SPY2 trial which focus on the BC patients with a high-risk and stage II/III evaluated pCR rates of pembrolizumab combined with neoadjuvant chemotherapy. Both NCT02513472 and NCT03051659 paid attention to the combination of pembrolizumab and eribulin. A summary of completed and ongoing Phase Ib/II and Phase III clinical trials in BC is presented in **Tables 1**, **2**.

**Table 1:** Summary of primary phase III clinical trials adding immunotherapy to chemotherapy in breast cancer.

Trial (National Clinical Trial Identifier)	Phase	Interventions	Patients enrolled	Number of patients	Primary endpoint	Key Results	Ref
**IMpassion130 (NCT02425891**)	III	Nab-paclitaxel ± atezolizumab	Untreated metastatic TNBC	902 (451 treated with atezolizumab)	PFS	Median PFS 7.2 months VS 5.5 months(PD-L1+ 7.5 months)	(18)
			unselected for PD-L1		OS	Median OS 21.3 months VS 17.6 months (PD-L1+ 25.0months)	
**IMpassion131 (NCT03125902)**	III	Paclitaxel ± atezolizumab	Inoperable locally advanced/metastatic	651 (293 PD-L1+)	PFS	Median PFS 6.0 months VS 5.7 months(PD-L1+ 7.5 months)	(15)
			TNBC				
**IMpassion132 (NCT03371017)**	III	First-line chemotherapy (capecitabine [mandatory in platinum-pretreated patients] or gemcitabine+ carboplatin) ± atezolizumab	Early relapsing metastatic TNBC	approximately 350	OS	Ongoing	(19)
**Impassion031 (NCT03197935)**	III	chemotherapy (nab-paclitaxel +doxorubicin + cyclophosphamide) ± atezolizumab	Early-stage TNBC (untreated stage II–III)	333 (165 treated with Chemotherapy+ atezolizumab)	pCR	Ongoing at data cutoff (April 3, 2020)	(22)
						pCR 58% VS 41%	
						pCR 69% VS 49% (PD-L1+)	
**KEYNOTE-119(NCT02555657)**	III	pembrolizumab arms VS chemotherapy arms	mTNBC (treatment with anthracycline or taxane before)	622 (312 pembrolizumab)	OS(PD-L1 CPS>=1 or CPS>=10)	Median OS 10·7 months VS 10·2 months (PD-L1 CPS>=1)	(23)
						12.7months VS 11.6 months (PD-L1 CPS>=10)	
						9·9 months VS11.8 months (overall population)	
**KEYNOTE-355 (NCT02819518)**	III	chemotherapy (nab-paclitaxel; paclitaxel; or gemcitabine plus carboplatin) ± Pembrolizumab	Previously untreated locally recurrent inoperable or mTNBC	847 (566 pembrolizumab)	OS、PFS(PD-L1 CPS>=1 or CPS>=10 and ITT populations)	Median PFS 9·7 months VS 5·6 months(PD-L1 CPS>=10)	(20)
						7.6 months VS 5·6 months (PD-L1 CPS>=1)	
						7.5 months VS 5·6 months (ITT population)	
**KEYNOTE-522 NCT03036488**	III	Chemotherapy(paclitaxel +carboplatin) ± pembrolizumab	Early-stage TNBC (untreated stage II–III)	1174	pCR	first interim analysis	(21)
					EFS (ITT population)	pCR 64.8% VS 51.2%	
						the incidence of treatment-related adverse events of grade 3 or higher 78.0%VS 73.0%	

**Table 2:** Summary of phase Ib/II clinical trials adding immunotherapy to chemotherapy in breast cancer.

Trial (National Clinical Trial Identifier)	Phase	Interventions	Patients enrolled	Number of patients	Primary endpoint	Key Results	Ref
**NCT01633970**	Ib	Nab-paclitaxel ± atezolizumab	Stage IV or locally recurrent TNBC (all patients experienced at least 1 treatment-related adverse event)	33	safety	73% grade 3/4 adverse events,	(24)
					tolerability	21% grade 3/4 adverse events of special interest and no deaths	
**KEYNOTE-173 (NCT02622074)**	Ib	Pembrolizumab+ chemotherapy	Early-stage TNBC (high-risk)	60	safety	neutropenia adverse event 73%	(25)
					RP2D	Immune-mediated adverse events and infusion reactions 30%(grade>=3 10%)two cohorts meet the RP2D threshold	
**NCT02513472**	Ib/II	Eribulin +pembrolizumab	mTNBC(≤2prior systemic anticancer therapies in the metastatic setting.)	167	safety, tolerability	ORRs	(26)
					ORR	25.8% (stratum1 n=66)	
						21.8% (stratum2 n=101)	
						ORR PDL-1+ VS ORR PDL-1-:	
						34.5% VS16.1% (stratum 1)	
						24.4% VS 18.2% (stratum2)	
**ALICE (NCT03164993)**	II	Chemotherapy (pegylated liposomal doxorubicin+ cyclophosphamide) ± atezolizumab	mTNBC	75	Safety	Ongoing	(27)
					PFS		
**KEYNOTE-086 (NCT02447003)**	II	Pembrolizumab	Previously treated mTNBC (prior treatment with anthracycline and taxane)	170 (105 PD-L1+)	ORR	ORR 5.3%	(28)
					safety	(PD-L1+ 5.7%)	
**NCT03051659**	II	Eribulin ± pembrolizumab	HR+/ERBB2-metastatic breast cancer	88	PFS	median PFS 4.1 vs 4.2 months	(29)
**I-SPY2 Trial (NCT01042379)**	II	NACT (taxane and anthracycline) ± pembrolizumab	Early-stage breast cancer (high risk)	300	pCR	ongoing, estimated pCR rates	(30)
						pCR 44% vs 17% (ERBB2- cohort)	
						pCR 30% vs 13% (HR+/ERBB2- cohort)	
						pCR 60% vs22% (TNBC cohort)	
**GeparNuevo (NCT02685059)**	II	NACT (nab-paclitaxel + EC) ± pembrolizumab	Early-stage TNBC	174	pCR	pCR 53.4% VS 44.2%	(31)
**ICON (NCT03409198)**	IIb	Chemotherapy ± ipilimumab and nivolumab	Metastatic HR+ breast cancer	75	Safety	Ongoing	(32)
					PFS		

CPS, combined positive score; EFS, event-free survival; EC, E=epirubicin, C= cyclophosphamide; ERBB2-, ERBB2-Negative; HR+, Hormone Receptor Positive; ITT, intention-to-treat; ORR, objective response rate; OS, overall survival; PD-L1, programmed death-ligand 1; pCR, pathological complete response; PFS, progression-free survival; RP2D, recommended phase II dose; stratum 1, number of prior systemic anticancer therapies is 0; stratum 2, number of prior systemic anticancer therapies is 1–2; TNBC, triple negative breast cancer; mTNBC, metastatic triple-negative breast cancer; NACT, neoadjuvant chemotherapy.

## Enhancing the Antigenicity or Adjuvanticity of BC Cells

### Impact of Chemotherapy on Tumor Antigenicity

In recent years, in the absence of infection, a novel type of cell death has been shown to be capable of triggering CD8^+^ T cells-mediated responses against “dying cell” neoantigens through cell stress-related processes, which has become an emerging research interest and has been referred to as “immunogenic cell death” (ICD) (33, 34). Chemotherapy-mediated ICD is also governed by cell stress, where the involved fundamental processes are regulated by cytoprotective pathways such as autophagy and endoplasmic reticulum stress (35, 36). Evidence available indicated that obviously enhanced tumor antigenicity induced by chemotherapeutic drugs might be caused by elevated major histocompatibility complex (MHC) expression and presentation of tumor neoantigens (TNA) or tumor-associated antigens (TAA) (37). Many existing chemotherapeutic agents and ionizing radiation can enhance the tumor antigenicity and the adjuvanticity effects of malignant cells when they elicit ICD and anticancer immunity (38). Anthracyclines, the cornerstone of chemotherapy regimens for BC, have been proven to one initiator or potentiator of ICD process through activation of the NLRP3 inflammasome (39). Previous preclinical studies demonstrated that 5-fluorouracil (5-FU) directly induced the upregulation of membrane-associated carcinoembryonic antigen (CEA) and MHC molecules in BC cell lines (40). Docetaxel and doxorubicin were also shown to promote the expression of antigen-processing machinery components, resulting in increased loading of MHC-I molecules in BC cells (41). Topotecan characterized as topoisomerase I-targeting drug showed immunogenic potential in TNBC cells by stimulating MHC I expression, inducing the secretion of interferon-β and activation of type I IFN signaling (42). Furthermore, an increasing expression of antigen-presenting molecules (MHC-I, MHC-II, and CD1d) was observed after gemcitabine and cyclophosphamide treatment in 4T1 mammary carcinoma cells, and thus promoting the antigen presenting behavior of dendritic cells (DCs) (43–45). The elevated expression of MHC-II and CD86 mediated by novel chemotherapeutic compound was also reported in TNBC cell line MDA-MB-231 (46). There are clear associations between the presence of MHC molecules and clinical outcomes in BC (47). Higher expression of MHC class II (MHC II) pathway genes expressions might predict longer disease-free survival (DFS) and low risk of recurrence for TNBC patients (48). Collectively, the upregulation of MHC-related molecules could remodel the immunopeptidome of cancer cells after chemotherapy, and thus enhancing their antigenicity.

### Chemotherapy-Induced Alterations of Damage-Associated Molecular Patterns (DAMPs)

At late time point of cell death, tumor cells can transfer “eat me signals” to facilitate immune cells phagocytosis and tumor antigen presentation, resulting in the conversion of dying tumor cells to adjuvanted-endogenous tumor vaccines (49). The nature of DAMPs is the fundamentally dynamic responding to chemotherapy-elicited cell stress that involve in multifaceted influences on extra- and intracellular microenvironments (50). The release of DAMPs often reflects the re-expression of novel membrane-bound, secreted proteins and increased intracellular components, such as type I interferon and adenosine triphosphate (ATP) (51). Among them, high mobility group box 1 (HMGB1), calreticulin (CRT) and surface heat shock protein 90 (HSP90) have been recognized as key ICD-related DAMPs, which were reported to improve antigen uptake and presentation of DC cells, and assist the CD8+ T cells to exert antitumor activity (52–54). These DAMPs induced by chemotherapeutic drugs could promote a state of anti-tumor immunity. However, other studies showed that DAMPs such as HMGB1, CRT, and ATP were also involved in BC progression, metastasis, and drug resistance (55–57). So, DAMPs represent a double-edged sword in BC.

The interactions between HMGB1 and TLR-2, TLR-4, and TLR-9 could also participate in cross-presentation of anti-tumor T lymphocytes *in vivo*, which lead to the activation of DCs and trigger antitumor immune responses (58, 59). In BC patients, the expression of HMGB1 was able to effectively measure the immunogenicity and effectiveness of chemotherapeutic drugs (60). *In vitro*, the level of extracellular HMGB1 was increased in conditioned media after doxorubicin treatment in MB-231 cells (61). Moreover, a significant increase of HMGB1 release was also determined in HCC1143 cells with epirubicin/docetaxel intervention (62). After neoadjuvant chemotherapy (NCT), plasma HMGB1 dramatically increased for BC patients who apparently obtain complete pathological complete response or partial remission (62). Another report also demonstrated that upregulated expression levels of HMGB1 and CRT were found after NCT in both BC patients and cell lines. And increase levels of HMGB1 have been shown to predict an improved therapeutic outcome in BC patieants receiving NCT (63, 64). CRT is an essential initiator of ICD signaling that is exposed at the surface of membrane and surrounded by immature and mature DCs (54). In a BC model, docetaxel did not alter the secretion of HMGB1 or ATP. However, exposure to CRT was observed in BC cell lines after docetaxel intervention, and antitumor immunity was reinforced mainly by the increased antigen presenting capacity and translocation of CRT (41). *In vitro* studies indicated that paclitaxel, gemcitabine and doxorubicin-mediated chemotherapy could efficiently kill cancer cells and lead to a high level of DAMP (CRT and HMGB1) (65–67). It has been shown that cyclophosphamide analogues improved tumor immunogenicity by facilitating the release of ICD markers (CRT, HMGB1, and ATP) (43). Altogether, these observations underscore the importance of adjuvanticity for chemotherapy to support the initiation of clinically anti-tumor immunotherapy.

## Activation of Immune Effector Cells

### Impact of Chemotherapy on the Innate Immunity

Innate immune cells including DCs, natural killer (NK) cells and macrophages may at least represent as adjuvants to immune checkpoint inhibitors (68). Some chemotherapies drugs have direct implications for DCs and NK cells. *In vitro* studies showed NK cells-mediated cytotoxicity against BC cells was significantly enhanced following epirubicin-based pretreatment indicating the combination of anthracycline-based chemotherapy and NK cells-based immunotherapy was potentially an efficient strategy for BC treatment (69). Initially, cytotoxic chemotherapeutics were demonstrated to induce an overall dysfunction of NK cells responses in localized and metastatic BC patients (70, 71), while the NK cells (CD56) numbers and macrophages (CD14) rapidly returned to normal after adjuvant chemotherapy (72). Another study reported that both epirubicin-based and doxorubicin-based regimen could result in an increased percentage of monocytes and NK cells, but a marked decrease was observed in B-cell numbers (73). Similarly, advanced BC patients using single-agent paclitaxel or docetaxel led to an enhancement of NK and LAK cytotoxic activity and increase of IFN-γ, IL-2, IL-6, GM-CSF cytokine levels in serum (74, 75). For clinical practice, a reduction in the infiltration of NK cells into tumor tissue has been proposed to be a predictor of chemotherapeutic treatment failure in BC (76, 77). During follow-up after adjuvant therapy, a previous study reported that NK cells cytotoxicity showed significantly elevated at all time-points and did not correlate with the mode of adjuvant radiotherapy or chemotherapy after a one-year follow-up (78). In addition, other studies suggested that the absolute number of activated NK cells was higher in BC patients who achieved pathological complete responses (PCR) after neoadjuvant chemotherapy, which implied that the improvement of NK cell activities was essential requirement for pCR especially in HER2-positive BC patients (79, 80). NCT could induce immune activation and a release from local immunosuppression in the tumor microenvironment, and thus activation of peripheral NK cells might promote the elimination of metastatic tumors in BC (81).

The impacts of chemotherapy on DCs have also been studied in BC. The antitumor efficacy of chemotherapies drugs is essentially determined by DCs that present antigens to tumor-specific T lymphocytes (39). Paclitaxel and doxorubicin were shown to improve the antigen presentation ability of DCs through stimulating the expression of costimulatory molecules and IL-12p70 (82). A study found that DCs in tumor lysate could consistently activate CD8+ CTLs for killing cancer cells in locally advanced BC, indicating DC-based vaccinations might be well suited to treat chemotherapy-resistant BC patients (83). A combination of doxorubicin and cyclophosphamide with autologous DCs was favorable to prolong the survival of T cells and recover immune functions capacity (84). One mechanism might be that this combination enhanced tumor immunogenicity as cryptic vaccines and promoted the adjuvant effects of ICD. Additionally, a recent multi-omics analysis revealed that BC patients with higher level plasmacytoid DCs tended to exhibit a more sensitive immune response and chemotherapies response, which highlighted that the potential benefit from combination of chemotherapy and immunotherapy might be achieved in BC patients with high immune infiltration of plasmacytoid DCs (85). Regarding the associations between DCs and chemotherapy in the clinic, significant efforts have been made. Prior to NAC, a marked unresponsiveness to *in vitro* stimulus was observed for DCs, while NAC could induce a remarkable responsiveness of APC compartments (86). A previous study also described a correlation between circulating DCs level and pCR in BC and their findings suggested that patients with a poor pCR after NAC were characterized by low expression of myeloid-derived DCs and plasmacytoid DCs (87). Altogether, these observations pave the way to translate innate antitumor immunity into innovative immunotherapies for fighting refractory BC.

### Impact of Chemotherapy on the Adaptive Immunity

B cells displayed dramatic depletion after chemotherapy and remained persistent low level even 9 months following systemic chemotherapy (88, 89). It has been reported that the percentage of peripheral blood B cells was substantial decreased by FEC (5-fluorouracil, epirubicin, cyclophosphamide) or FDC (5-fluorouracil, doxorubicin, cyclophosphamide) regimens in BC (73). Likewise, vinorelbine, cyclophosphamide and 5-FU were also reported to decrease the number of circulating B cells in which cyclophosphamide had the largest influence over levels of B cells (90). The reason for cytotoxic chemotherapy effect on B cells was partly due to an increased sensitivity of B cells to chemotherapeutic agent *in vitro* compared to T-cells (91). Tumor infiltration of B cells in the tumor microenvironment could serve as a promising biomarker to select BC patients who might benefit from NAC (92). Memory B cells was correlated with pCR to NAT in ER-negative BC tumors, which indicated humoral immunity was essential for mediating response to cytotoxic therapy (93). Also, higher B cells infiltration could potentiate the local cytotoxic immune response and were correlated with better outcomes in hormone receptor-negative BC patients (94).

Substantial evidence suggested that chemotherapy contributed to T-cells independent immune responses. *In vivo* treatment of tumor-bearing mice demonstrated that doxorubicin led to a significant increase in the number of CD4 + T cells, CD8+ T cells and NK cells and promoted expression of interferon γ (IFN-γ) and granzyme B (95). In another pre-clinical experiments, the administration of anthracycline also facilitated the infiltration of CD4+ and CD8+ T cells in TNBC mouse model (96). Several possible mechanisms have been proposed to explain these phenomena. Treatment of doxorubicin promoted cytotoxic T lymphocytes accumulation by a potent production of IFN-γ and IL-17 in a BC mouse model, which suggested that γδ T cells indeed played a sizable role in doxorubicin-induced anti-tumor immune response (97). Low doses of cyclophosphamide were shown to reverse the immunosuppression and strongly enhanced the abundance of tumor infiltrating T cells *via* the secretion of various cytokines and activation antigen-presenting cells (98). Furthermore, high dose of cyclophosphamide could completely eradicate tumor cells, while cyclophosphamide at low doses was able to reduce the number of circulating Tregs but increase the production of tumor-specific T cells (99). In clinical contexts, the percentages of CD3+, CD4+ T cells and Treg cells in blood samples of BC were significantly decreased after 6 cycles of chemotherapy (100). To assess the effect of combination chemotherapy on subsets of immune cells, a study revealed that anthracycline-based regimen could induce an increase of cytotoxic T and NK cells, but a dramatic decrease of B cells in blood (73). A better clinical response during chemotherapy has been linked to higher level of circulating CD8+ T-cell (101). Some studies have addressed the effects and correlations of NAC on effector T cells. After NAC, BC patients with beneficial therapeutic effects often correlated with an increased level CD4+ and CD8+ T-cells, and decreased CTLA-4+ T cells and VEGF (102, 103). It has been previously documented that the expression of CD8/Foxp3 was upregulated in cancer tissues of pCR cases, which implied that activation of antitumor T cell responses was occurred in these tumors (104). Tumor microenvironment characteristics analysis further revealed that higher level of stromal tumor infiltrating CD8+ T cells and B cells significantly correlated with pCR in NAC (105–107). However, existing studies have focused on the prognostic value of infiltrating of immune effector cells on chemotherapy. Understanding how to maximize the therapeutic potential of chemotherapy-induced immunomodulatory effects remains an open question.

## Hampering the Functions of Immunosuppressive Cells

### Treg Cells

Treg cells mainly function in preventing excessive immune activation. Blocking or depleting Tregs is therefore a viable therapeutic strategy to enhance antitumor immunity (108). Studies have revealed that the depletion of Treg cells in immune cell infiltrate was associated with a protective anticancer immunity. This also meant that anticancer immunity switched from a silent immune response to an active immune response (109, 110). A study showed that BC patients had more Treg cells than normal individuals. Meanwhile, an increasing level of Treg cells and lower ratio of Th/Tr cells were found in Stage IV BC patients compared to stage I, II, or III BC patients (111). It has been described that the percentage of Treg cells was reduced after 6 chemotherapy cycles among stage II/III BC patients (100). Paclitaxel was shown to not only reduce CD4+Foxp3+ Tregs cells but hinder cytokine production of Tregs (112). The weakening effect of cyclophosphamide on Tregs cells was often observed at low dose (99). Additionally, metronomic cyclophosphamide regimens also led to a profound and effective Treg inhibition in metastatic BC patients (99). Low Treg abundance was determined in TNBC but not in ER-positive or Her2-negative subtype, especially for patients with pCR after NAC, which indicated that Treg abundance might serve as a predictive biomarker for evaluating their NAC effectiveness in TNBC (113).

### M2 Macrophages and MDSCs

Tumor-associated M2 macrophages (M2-TAMs) was proposed to promote immune escape and limit the efficacy of immunotherapy. Targeting M2-TAMs synergizes with immune checkpoint blockade has emerged as promising strategies for cancer treatment (114). Docetaxel administration could induce a switch from M2-like phenotype to M1-like phenotype in mammary tumor-bearing mice (115). In another 4T1 BC lung metastasis mice model, nanosystem-based co-delivering doxorubicin was also able to modulate the polarization from M2 macrophages to antitumor M1 macrophages (116). BC patients who fail to respond to anthracycline-containing NAC were predominantly associated with the presence of M2+ macrophage phenotype (117).

Myeloid-derived suppressor cells, a heterogenic population of immature myeloid cells, were characterized by their immunosuppressive effects. Cytotoxic agents against MDSCs represent therefore an appealing therapeutic strategy for cancer therapy but its underlying molecular mechanism remains obscure (118, 119). So far, many cytotoxic chemotherapeutics were shown to have excellent repression on MDSCs in BC (120). In mouse model of BC, an inhibitory effect on MDSC of doxorubicin has been demonstrated in the spleen, blood, and tumor tissues (95). Furthermore, the treatment of doxorubicin could increase the frequency of the effector lymphocytes or NK cells that effectively reduced MDSC ratios (95). The above studies not only suggested the direct cytotoxic effect on cancer cells, but also highlighted the immunomodulatory role of doxorubicin on MDSC. In another animal models, downregulation of splenic CD44+, IL-17A+ MDSCs effect of cisplatin was revealed by single cell mass cytometry in 4T1 metastatic BC model (121). Docetaxel, one chemotherapeutic agent for treating anthracycline-refractory BC, have been reported to suppress the level of MDSCs and stimulate the CTL response in spleens of mice (115). Gemcitabine and cyclophosphamide were also found to be capable of inhibiting the accumulation of MDSCs (43). Beyond that, capecitabine depleted MDSCs and relieved their inhibitory effects on T and NK cells (122). A single arm, pilot study observed that levels of circulating MDSCs increased after doxorubicin and cyclophosphamide treatment but decreased after paclitaxel treatment for BC patients with NAC (123). Compared to patients with Non-pCR following NAC, circulating MDSCs seemed to lower for complete or near pCR BC patients (123). Additional studies have also demonstrated that BC patients with a lower level of circulating MDSCs before treatment preferred to achieve a higher probability of a pCR after the last cycle of chemotherapy (124). However, it is a well‐recognized challenge to determine the target against MDSCs owing to its multiface of MDSCs and the complexity of tumor microenvironment. Besides, considerable research efforts are focusing on the total MDSCs populations in BC. Thus, the immunomodulatory effects of chemotherapy on different MDSC subtypes remain to be explored.

### Effects of Anticancer Agents on the Immune Checkpoints

In the past, BC was thought to be a “cold” tumor with low immunogenicity and mutation burden. However, studies in recent years have identified high PD-L1 and tumor infiltrating lymphocytes in TNBC and HER-2-positive breast cancers (125, 126). At the preclinical level, doxorubicin was shown to inhibit tumor immunosuppression through down-regulating the expression of immune checkpoints PD-1 and TIM-3 in the tumor tissue (127). In a TNBC murine model, doxorubicin/cyclophosphamide regimen was able to effectively inhibit tumor growth, increase the survival benefit, promote infiltrating of CD8+ T cells and suppress the suppressor molecules PD-L1 expression (128). With regard to PD-L1 expression changes in BC after chemotherapy, a panel of six anti-cancer compounds were experimentally found to induce PD-L1 expression in four BC cell lines through a cellular stress response pathway (129). Study by Samanta et al. demonstrated that doxorubicin, gemcitabine, or paclitaxel induced HIF-dependent, transcriptional activation of CD47, CD73, and PDL1 expression that imparted TNBC cells the ability to evade the immune systems (130). Similar findings have been reported that paclitaxel, etoposide and 5-fluorouracil could induce PD-L1 expression in BC cells and up-regulated PD-L1 promoted PD-L1-specific T cell apoptosis (97). After treating with metronomic cyclophosphamide, BC patients exhibited a higher expression PD-L1 in tumor cells; however, no obvious benefit was observed for CTX regimens combined with concomitant PD-L1 antibody therapy (131). A case report described that level of CD8 and PD-L1 expression on immune cells were increased after capecitabine and gemcitabine-carboplatin-iniparib therapy (132). A clinical trial aimed to identify molecular alterations of immune gene signatures following neoadjuvant chemotherapy of TNBC and they found several immune checkpoints including IDO1, PD-L1 and CTLA4 were upregulated in pre-treatment samples who achieved pCR (133). Collectively, the absence of unifying PD-L1 protocols makes it hard to draw a convincing conclusions from these studies. Besides, PD-L1 levels are generally evaluated in tissues prior to chemotherapy, which might not reflect the real status of the tumor microenvironment after chemotherapy.

## Conclusion

**Figure 1:**
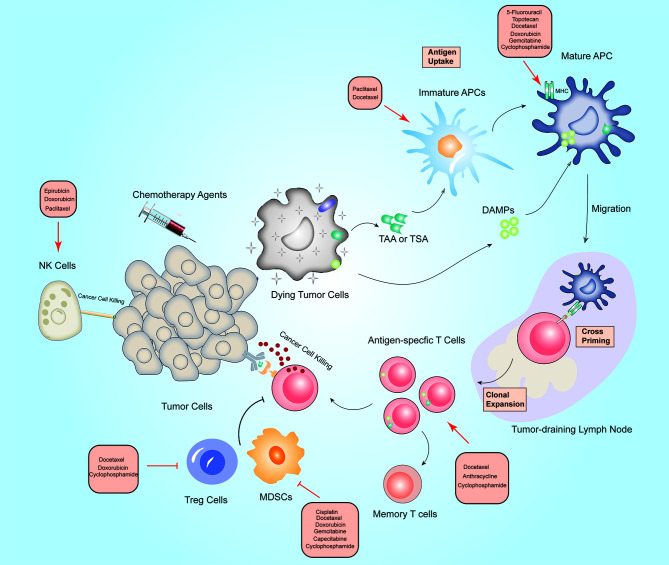
Overview of the immunostimulatory properties of chemotherapy in breast cancer. On-target effects: When tumor cells are exposed to chemotherapeutic drugs, TAA, TSA and DAMPs release by dying tumor cells are engulfed by immature DCs, which promotes APCs maturation. Archived antigen-bearing APCs then migrate to the tumor-draining lymph node, where APCs cross-prime to T cells. Thereafter, antigen-specific T cells undergo clonal expansion, and at least some of them differentiate into memory T cells. Activated T cells then recognize tumor cells and mediate cytotoxic killing of tumor cells. Off-target effects: Chemotherapeutic drugs can activate immune effector cells including natural killer (NK) cells, dendritic cells (DCs), and cytotoxic T cells, and depletion of immunosuppressive cells including Treg cells, M2 macrophages and (myeloid-derived suppressor cells) MDSCs. Red arrows indicate an increased effect and red flat ended lines represent an inhibitory effect. The text boxes near the arrows list the chemotherapy agents that elicit immunomodulatory effects in BC.

For many decades, cytotoxic chemotherapeutics are still the cornerstone of BC treatment (134). However, encouraging advancements in cancer immunotherapy have provided more options for certain subtypes of BC (11, 135). Single chemotherapeutics agents or single immuno-oncological therapy cannot obtain ideal therapeutic effect for advanced BC (136). Thus, combining immunotherapy with the currently-available therapies has shown great promise. Current mini-review summarizes the updated clinical trials on immunotherapy and chemotherapy combinations in BC (**Tables 1**, **2**) and provides an overview of immune-stimulating properties of cytotoxic chemotherapy (**Figure 1**). There remains large room for improvement of synergistic effects of these two combined modalities, so identifying prerequisites for designed immunotherapy combination strategies are of special importance.

ICD is a specific type of cancer cell death characterized by antigen-specific immune responses against the antigens of dying cancer cells (137). Anthracycline and taxanes-containing chemotherapy can promote immunostimulatory activity by increasing the antigenicity or adjuvanticity of cancer cells (138). The ICD effects mediated by chemotherapy have largely centered on chemotherapy-induced alterations of DAMPs (50, 139). Notably, through DAMPs mechanisms, chemotherapy stimulates immune system to recruit DCs and activate the immune responses specific for tumor-relevant antigens. Conversely, fewer studies have looked at the effects of chemotherapeutic drugs on tumor cell antigenicity. Future studies are required to elucidate the molecular mechanism of DAMPs in ICD and provide specific interventions targeting them to facilitate development of chemoimmunotherapeutic regimens. In BC, numerous studies have demonstrated that chemotherapeutic agents can act directly on immune cell subsets to elicit antitumor immunity. Off-target effects of chemotherapy on immune cell subsets mainly involve activation of immune effector cells including NK cells, DCs, and CTLs, and depletion of immunosuppressive cells including Treg cells, M2 macrophages and MDSCs. However, the dynamic alterations of effector immune cells in full course of adjuvant chemotherapy remain unknown.

Cytotoxic chemotherapies may act as upfront measures that are capable of converting “cold” BC tumors into “hot” lesions, which may be successful clearance with ICIs. In the present review we have focused on the immunomodulatory effects of chemotherapy in BC. In addition to chemotherapy, endocrine therapy, targeted therapeutic agents and radiation have also been demonstrated to have analogous immunoregulatory function for BC, in particular for radiotherapy (140, 141). Thus, these therapeutic options should also be suggested for combined immunotherapy based on different intrinsic subtypes of BC. The immunotherapy era provides additional selections for clinicians in BC treatment, but at the same time, many unanswered questions exist regarding combinations with chemotherapy and immunotherapy. How to identify prerequisites of combination treatment given patient’s immune status and intrinsic characteristics. Limited information is available on the impact of cytotoxic chemotherapy on immune checkpoints pathways not confined only PD-L1, PD-1 or CTLA4. Lastly, it should be noted that single-agent chemotherapy can act on multiple steps of antitumor immune response, and one chemotherapy regimen may also play two opposite roles in different immune targets.

Therefore, when considering potential applications in clinic, drug dose, timing of administration and appropriate population would need to be carefully considered.

## Author Contributions

XH and JZ were involved in the design of the work and figures. JZ and SP performed the literature search and wrote the draft. CJ, LH, JD, QS and HJ edited the manuscript and provided the critical revisions. All authors contributed to the article and approved the submitted version.

## Funding

This work was supported by grants from the Science and Technology Program of Anhui Province (1804 h08020259). and Scientific Research Start-up Funds of The First Affiliated Hospital of USTC ( RC2021122).

## Conflict of Interest

The authors declare that the research was conducted in the absence of any commercial or financial relationships that could be construed as a potential conflict of interest.

## Publisher’s Note

All claims expressed in this article are solely those of the authors and do not necessarily represent those of their affiliated organizations, or those of the publisher, the editors and the reviewers. Any product that may be evaluated in this article, or claim that may be made by its manufacturer, is not guaranteed or endorsed by the publisher.
